# Retroviral insertional mutagenesis implicates E3 ubiquitin ligase RNF168 in the control of cell proliferation and survival

**DOI:** 10.1042/BSR20170843

**Published:** 2017-08-14

**Authors:** Aytug Kizilors, Mark R. Pickard, Cathleen E. Schulte, Kiren Yacqub-Usman, Nicola J. McCarthy, Shu-Uin Gan, David Darling, Joop Gäken, Gwyn T. Williams, Farzin Farzaneh

**Affiliations:** 1 Department of Haematological Medicine, King’s College London, The Rayne Institute, London SE5 9NU, U.K.; 2 Apoptosis Research Group, School of Life Sciences, Huxley Building, Keele University, Keele ST5 5BG, U.K.; 3 Institute of Medicine, Bache Hall, University of Chester, Chester CH2 1BR, U.K.; 4 Tumour and Vasculature Biology, School of Medicine, University of Nottingham, Queen’s Medical Centre, Nottingham NG7 2UH, U.K.; 5 Horizon Discovery Ltd, 7100 Cambridge Research Park, Beach Road, Waterbeach, Cambridge CB25 9TL, U.K.; 6 Department of Surgery, National University of Singapore, Singapore 117597

**Keywords:** Retroviral Insertional Mutagenesis, Ubiquitin, Apoptosis, Cell survival, Cell proliferation

## Abstract

The E3 ubiquitin ligase RNF168 is a ring finger protein that has been previously identified to play an important regulatory role in the repair of double-strand DNA breaks. In the present study, an unbiased forward genetics functional screen in mouse granulocyte/macrophage progenitor cell line FDCP1 has identified E3 ubiquitin ligase RNF168 as a key regulator of cell survival and proliferation. Our data indicate that RNF168 is an important component of the mechanisms controlling cell fate, not only in human and mouse haematopoietic growth factor dependent cells, but also in the human breast epithelial cell line MCF-7. These observations therefore suggest that RNF168 provides a connection to key pathways controlling cell fate, potentially through interaction with PML nuclear bodies and/or epigenetic control of gene expression. Our study is the first to demonstrate a critical role for RNF168 in the mechanisms regulating cell proliferation and survival, in addition to its well-established role in DNA repair.

## Introduction

The expression, replication and repair of DNA are pivotal to the physiological functioning of all cells. Understanding of the molecular mechanisms, control and integration of these processes is far from complete and this area is the subject of a great deal of research. This is justified, not only by the obvious importance of these processes in cellular physiology, but also by the many examples of abnormalities in these processes contributing to oncogenesis (reviewed by Hanahan and Weinberg [1]). The existence of important connections between DNA damage sensing mechanisms (DNA damage response (DDR)) and control of gene expression and their effect on processes such as cell proliferation and differentiation have been suspected for several decades [2]. There is now direct molecular evidence for some of these mechanisms. These range from DNA strand break mediated activation of p53 inhibiting haematopoietic progenitor cell differentiation [3,4], and the induction of differentiation by specific DDR genes such as *GADD45A* in haematopoietic stem cells [5], to the involvement of CDK12 in the regulation of DDR and embryonic development [6] as well as damage-induced modulation of miRNAs that affect cell cycle progression, apoptosis and differentiation [7–9] .

Ongoing progress in our understanding of gene expression, DNA replication and repair most often relies on detailed investigation of previously identified molecules and, as a consequence, generally progresses incrementally. By contrast, forward genetics strategies allow unbiased approaches that can identify key molecules involved in rate-limiting steps independently through the subversion of individual gene function [10]. Successful forward genetics strategies include cDNA functional expression cloning [11–16] and retroviral insertional mutagenesis (RIM) [16–20]. Indeed, current RIM studies have focused attention on the role of E3 ubiquitin ligase RNF168 in the control of cell fate.

Post-translational modification of proteins is extensively involved in controlling cell behaviour. Addition of ubiquitin to target proteins, either as a monomer or in the form of ubiquitin chains, is now recognized to have many important regulatory roles in addition to the targeting of proteins for degradation by the proteasome [21,22]. In particular, ubiquitination of nuclear proteins plays a central role both in DNA repair [22–24] and in epigenetic control of gene expression [25–27], including the expression of tumour suppressor genes [27].

Extensive studies have implicated RNF168 in the repair of double-strand DNA breaks [23,28–32]. The repair of double-strand DNA breaks is a complex process in which RNF168 and RNF8 catalyse the ubiquitination of histone H2A subtypes that leads to recruitment of protein components of the DNA repair machinery, including 53BP1 and BRCA1 [28–32]. Mutation in RNF168 produces RIDDLE syndrome in humans [33], although some of the features of the phenotype, such as craniofacial abnormalities and short stature, have hitherto been difficult to ascribe to aberrant DNA repair alone.

Although *RNF168* is amplified in some cancers [32,34], the observations reported below are the first to demonstrate the involvement of this gene in the control of cell survival and proliferation. Most recently, RNF168 has been shown to regulate PML nuclear bodies (PML NBs) [35], suggesting a potential mechanism for the regulation of proliferation and apoptosis by RNF168 described below.

## Materials and methods

### Materials

Recombinant mouse interleukin-3 (mIL-3) was obtained from R&D Systems (Abingdon, U.K.) and recombinant human interleukin-3 (hIL-3), reagents for real-time quantitative RT-PCR (RT-qPCR), Lipofectamine 2000 and the pcDNA3.1 and TopoPCR2.1 vectors were from Life Technologies Ltd (Paisley, U.K.). Cell culture reagents were from the latter source or from Sigma–Aldrich (Poole, U.K.). The plasmid pCMVSPORT6-RNF168 (MGC: 45398; IMAGE 5163887), which contains the complete coding sequence of human RNF168, was from Source BioScience (Nottingham, U.K.) and nucleofector solution T was from Lonza Bioscience (Verviers, Belgium). QuikChange® XL Site-directed Mutagenesis Kit was from Agilent Technologies (Stockport, U.K.) and polybrene was from Sigma–Aldrich (Poole, U.K.). siRNAs #1–#4 to human RNF168 (product codes: #1, Hs_FLJ35794_1; #2, Hs_RNF168_2; #3, Hs_FLJ35794_3; #4, Hs_RNF168_1) were from Qiagen Ltd (Crawley, U.K.); negative control (NC) siRNA (product 102728) and HiPerFect reagent were also from the latter source. The MTS assay kit (CellTiter 96 AQueous One Solution Cell Proliferation Assay) was from Promega (Southampton, U.K.) and the Muse Cell Cycle Assay Kit was from Millipore (U.K.) Ltd (Watford, U.K.). Protein Assay Kit II and precast gels were from Bio–Rad Laboratories (Hemel Hempstead, U.K.). The RNF168 and β-actin antibodies for immunoblotting were from Abcam (Cambridge, U.K.), whereas the anti-myc and FITC-labelled anti-mouse IgG antibodies for immunofluorescence were from Santa Cruz Biotechnology (Heidelberg, Germany) and Sigma–Aldrich (Poole, U.K.) respectively. Hybond-P PVDF membranes were from Amersham Biosciences (Little Chalfont, U.K.).

### Cell culture

The mouse haematopoietic granulocyte/macrophage progenitor cell line FDCP1 [36–38] was maintained in RPMI-1640 medium supplemented with 10% FBS, 2 mM L-glutamine, 100 U/ml penicillin, 100 μg/ml streptomycin and 1 ng/ml recombinant mIL-3. Cells were deprived of mIL-3 by centrifugation and resuspension in mIL-3-free medium for two cycles of washing and cloning in soft agar without mIL-3. 293T cells were maintained in DMEM medium containing 10% FBS, 100 U/ml penicillin and 100 μg/ml streptomycin. TF-1 cells were routinely maintained in R-10 medium (comprises RPMI-1640 containing 2 mM L-glutamine, 1 mM sodium pyruvate, 10 mM HEPES, 10% FBS and 50 µg/ml gentamicin) supplemented with recombinant hIL-3 (5 ng/ml) and MCF7 [39] cells were maintained in R-10 medium; all cells were cultured at 37°C in a humidified incubator with 5% CO_2_.

### Generation of IL-3-independent FDCP-1B cell clones by RIM

In the present study, the PAPM3P packaging cell line which produces M3Pneo-sup virus was used for infection of FDCP1B cells. For RIM, a total of 1 × 10^8^ cells were co-cultured with the irradiated PAP3MP packaging cells for 2 days in the presence of mIL-3. Then the cells were harvested and incubated overnight in fresh medium also in the presence of interleukin-3 (IL-3). Next, while 1 × 10^8^ cells were seeded in soft agar in the absence of mIL-3, 1× cells were taken through to the next round of co-culture with the PAP3MP packaging cell line. These cycles of co-culture were repeated for a total number of 15 times. After each cycle, 1 × 10^8^ cells were cloned in soft agar in the absence of mIL-3. A total of 95 IL-3 independent FDCP1B clones were isolated at the end of 15 rounds of co-culture. The IL-3 independent FDCP1B clones were initially expanded in 1 ml culture in the absence of mIL-3 and G418. Following their successful expansion, G418 selection was performed on all clones, each of which was found to be G418 resistant. In the present study, we have analysed one of these clones, referred to as PAP60 [40].

### Inverse PCR

The inverse PCR (I-PCR) method was performed as described previously [41]. Briefly, genomic DNA of PAP60 cells was digested with the Sau3A restriction enzyme, followed by ligation and subsequent XbaI digestion. The sequence flanking the 3′-LTR of the integrated provirus in PAP60 was obtained using the outward directed primers MPSVA1 (AAACTGCTGAGGGCGGGACC) and MPSVA2 (AGTTCGCTTCTCGCTTCTGT). The PCR fragments produced by I-PCR were cloned into the TopoPCR2.1 vector and the genomic DNA at the 3′ end of the integrated provirus flanking sequence was sequenced, i.e. 5′-GTAATTTCTTCTTTTAGCACTAAGAACTTTAGAAAGCTTTG TTAGGAGAAGGGTAGCCTAAAGAATACTGAAAGAATAATTACAAAAATTCCTGTTCGGATC-3′ and identified by a BLAST search of the human genome.

### Plasmids and transfection

#### Transduction into FDCP1B

All pLKO.1-shRNA plasmids were designed by The RNAi Consortium with the following clone reference numbers: control shRNA targeting GFP (shRNA-eGFP (SHC005)) and shRNA targeting mouse RNF168; shRNF168A (TRCN0000040876), shRNF168B (TRCN0000040873) (Open BioSystems).

The coding sequence for full-length human RNF168 (IMAGE: BC046815) was subcloned into pcDNA3.1 plasmid in frame with a C-terminal Myc and His fusion domain; RNF168WT. The point mutations in the RING (RNF168H33A) and MIU domains (RNF168A179G/A450G) were introduced by site-directed mutagenesis using the QuikChange® XL Site-directed Mutagenesis Kit. All constructs were sequence verified.

#### Transfection into TF-1 cells

TF-1 cells (2 × 10^6^) in 0.1 ml nucleofector solution T, were nucleofected with 2 µg plasmid constructs comprising pCMVSPORT6-RNF168 or empty pCMVSPORT6 vector (for controls) using program G-016, and cells were immediately plated in 3 ml I-20 medium (comprises Iscove’s modified Dulbecco’s medium containing 2 mM L-glutamine, 20% FBS and 50 μg/ml gentamicin) supplemented with hIL-3 (5 ng/ml) and allowed to recover for 20 h. Cells were then washed twice with R-10 medium, and resuspended (3 × 10^5^ cells/ml) in R-10 medium ± hIL-3 (5 ng/ml) and cultured for a further 22 h before counting.

### RNAi

#### shRNA (LVshRNA)

Lentiviral vector was produced by transient co-transfection of 293T cells with lentiviral packaging plasmids; pCMVΔ8.91 and pMDG2 with the target lentiviral constructs; pLKO.1-shRNAs, using a calcium phosphate co-precipitation method. Supernatant, containing the vector, was harvested at 48  h (harvest I) and 72  h (harvest II). The vector supernatant was first clarified through a 0.45- μm filter and concentrated by centrifugation at 10000***g*** (Beckman J2-MC) overnight at 4°C, resuspended in 500 μl RPMI-1640 medium and stored at –80°C. Cells were infected with lentivirus vectors in the presence of 4 μg/ml polybrene. A multiplicity of infection (MOI) of 3 was used for infection.

#### siRNA

To determine the effect of RNF168 knockdown on culture growth, four siRNAs which target different portions of RNF168 sequence were individually studied, along with NC siRNA for controls. For TF1 cells, complexes were prepared by mixing 9 μl HiPerFect reagent with 209 µl siRNA (430 nM in Opti-MEM-I). After 15 min, these were added dropwise to cells (4 × 10^5^ cells in 0.2 ml R-10 medium with 5 ng/ml IL-3; 12-well plates). After a further 3 h, R-10 medium with 5 ng/ml IL-3 (0.8 ml) was added, and cells were cultured for a further 70 h to allow silencing to occur. Cells were then reseeded (3 × 10^5^ cells/ml in R-10 medium with 5 ng/ml hIL-3) and cultured for a further 72 h to assay the growth parameters. For MCF7 cells, a fast-forward transfection protocol was employed: complexes were prepared by mixing 12 μl HiPerFect reagent with 100 μl siRNA (120 nM in Opti-MEM-I). After 15 min, these were added dropwise to freshly trypsinized, exponentially growing cells (1.5 × 10^5^ cells in 2.3 ml R-10 medium; six-well plates) and these were cultured for 70 h. Cells were then replated in either fresh R-10 medium for MTS assay (0.5 × 10^4^ cells/96-well plate), direct cell counting and/or cell cycle analysis (both at 0.8 × 10^5^ cells/12-well plate) at the indicated times, or in R-10 medium supplemented with 10% cell-conditioned medium (400 cells/six-well plate) for clonogenic assays.

### RT-qPCR

Total RNA was isolated using the TRIzol® reagent and 1 μg of purified total RNA from each sample was reverse transcribed to cDNA with oligo-dT primers by using a SuperScript II™ Kit according to the manufacturer’s protocol. Quantitative PCRs were performed with Platinum® SYBR® Green qPCR SuperMix-UDG Kit (Thermo Fisher Scientific) using an Applied Biosystems 7900 HT thermal cycler. The following primers were used: mouse RNF168 (5′-AGGCAGGTCTGAGGAGAAAGTGTT-3′, 5′-AGGCAGGTCTGAGGAGAAAGTGTT-3′), mouse Smco1 (5′-ACCATGGAACTGAATATTGTATACAGCTAT-3′, 5′-AACTGCTGGGTCAAAGGTAAC-3′) and β-actin (5′-TGAACCCTAAGGCCAACCGTGAAA-3′, 5′-AGTCCATCACAATGCCTGTGGTACG-3′). Cycling conditions were 5 min at 95°C followed by 40 cycles of 15 s at 95°C, 30 s at 55°C and 30 s at 72°C. The Δ*C*_T_ method was applied to estimate relative transcript levels. Levels of β-actin amplification were used as an endogenous reference to normalize each sample.

### Growth analyses

Direct cell counting was performed after Nigrosin Blue (0.1% (w/v)) staining using a haemocytometer and light microscopy; cells which excluded the dye were considered to be viable. MTS assays were performed according to the manufacturer’s instructions. For clonogenic assays, the colonies formed following 3 weeks of culture were counted after staining with Crystal Violet (0.5% (w/v) in methanol). Cell cycle analysis was performed on a Muse Cell Analyzer using a Muse Cell Cycle Assay Kit according to the supplied instructions.

### Immunofluorescence

293T cells were cultured on glass coverslips in six-well plates and transfected with pcDNA3.1/RNF168WT, RNF168H33A or RNF168A179G/A450G plasmid using Lipofectamine 2000 according to the manufacturer’s instructions. Thirty-six hours after transfection, cells were fixed in 4% paraformaldehyde for 30 min and permeabilized in 0.2% Triton-X 100 solution for 15 min followed by blocking with 3% BSA/PBS for 30 min. For RNF168 protein detection by immunofluorescence, cells were incubated with mouse anti-myc primary antibody (1:200 dilution) for 1 h, after extensive washing, the cells were incubated with secondary antibody (FITC anti-mouse IgG, 1:100) for 1 h, followed by washing with PBS. Coverslips were air dried and counterstained with DAPI. Images were acquired by fluorescence microscopy.

### Western blot analysis

Whole protein lysates were extracted using RIPA lysis buffer supplemented with PMSF and protease inhibitor cocktail; the concentration of isolated proteins was determined using Protein Assay Kit II. Protein (50 μg) was electrophoresed (10% precast gel), then transferred to Hybond-P PVDF membranes. These membranes were incubated with anti-RNF168 in 5% skimmed milk and appropriate secondary antibodies. Blots were then stripped and reprobed with an antibody to β-actin. Western blot imaging and quantification were carried out using the LI-COR ECL system (LI-COR, Lincoln, U.S.A.).

### Statistical analyses

Data are presented as the mean and S.E.M.; the number of observations (*n*) refers to different transfected samples, each derived from a separate culture of cells. Data were analysed by either one-way ANOVA, using either Bonferroni**’**s multiple comparison test (MCT) or Dunnett’s MCT (the latter when comparing multiple groups compared with a single group) for post hoc analysis, or by two-way ANOVA, using Dunnett’s MCT. Homogeneity of variance was checked by Bartlett’s test and, where necessary, data were transformed (log or square root) prior to analysis. All analyses were performed using GraphPad Prism v4.03.

## Results

### RIM of mouse haematopoietic cells

The FDCP1 cell line is a growth factor dependent haematopoietic granulocyte/macrophage cell line originally isolated from DBA/2 mouse bone marrow [36]. FDCP1 cells were cloned using soft agar and limiting dilution in the presence of IL-3 and the subclone FDCP1B was selected for subsequent analyses because this subclone undergoes apoptosis more rapidly and synchronously on withdrawal of IL-3 [37] than parental FDCP1 cells [38].

**Figure 1:**
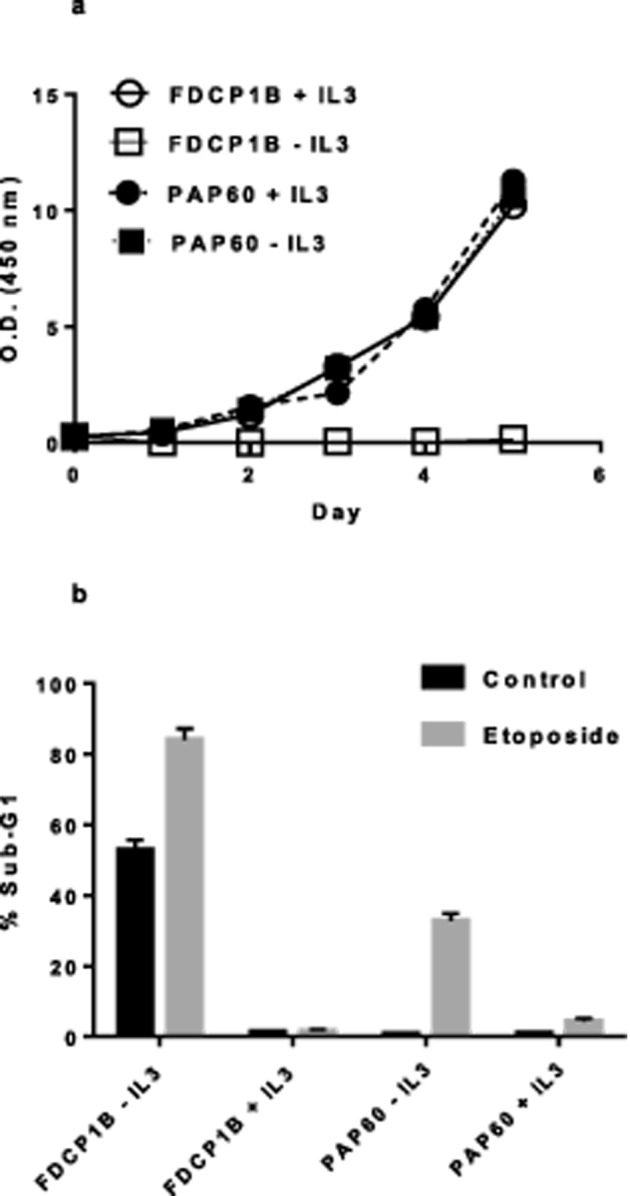
RIM clone PAP60 is IL-3 independent for both survival and proliferation but is not insensitive to IL-3 (**a**) FDCP-1B and PAP60 cells were washed three times with RPMI-1640 medium lacking mIL-3 and then seeded at 1 × 10^5^ cells per ml in the presence or absence of 1 ng/ml mIL-3. Cell proliferation was measured by MTT assay at the indicated time points over a 5-day period. (**b**) The presence of IL-3 still exerts a strong protective affect for FDCP-1B and PAP60 cells when apoptosis is induced by etoposide treatments. FDCP-1B and PAP60 cells were washed and seeded at 2 × 10^5^ cells in 2 ml culture medium, in the presence or absence of mIL-3 with 3.4-μM etoposide (Eto). After 24 h, cells were harvested, stained with Propidium Iodide, and apoptosis was detected using flow cytometry. Apoptotic bodies appear in the sub-G_1_ peak. Data are the mean ± S.D. of three independent experiments.

FDCP1B cells were infected with retrovirus M3Pneo-sup. M3Pneo-sup is a myeloproliferative sarcoma virus (MPSV)-based retroviral vector in which all the viral genes have been removed (splice acceptor sequences and upstream sequences necessary for efficient splicing of Mo-MuLV are retained) and the selectable marker gene, neomycin phosphotransferase (*neoR*) has been inserted in order to select cells that carry an integrated provirus [42–44]. The retrovirally infected FDCP1B cells were plated in soft agar in the absence of IL-3 and IL-3-independent clones were isolated. One of these clones, PAP60 [40], was selected for further study and showed a complete IL-3 independence for both survival and growth (Figure 1a). In order to determine whether the mutant cells could respond to IL-3 at all, they were challenged with topoisomerase II inhibitor etoposide in the presence and absence of IL-3 (Figure 1b). The presence of IL-3 produced substantial protection against etoposide-induced apoptosis of both the PAP60 mutant cell line and the parental cell line, indicating that the PAP60 cell line retained responsiveness to IL-3 even though it was no longer IL-3-dependent for survival or proliferation.

**Figure 2:**
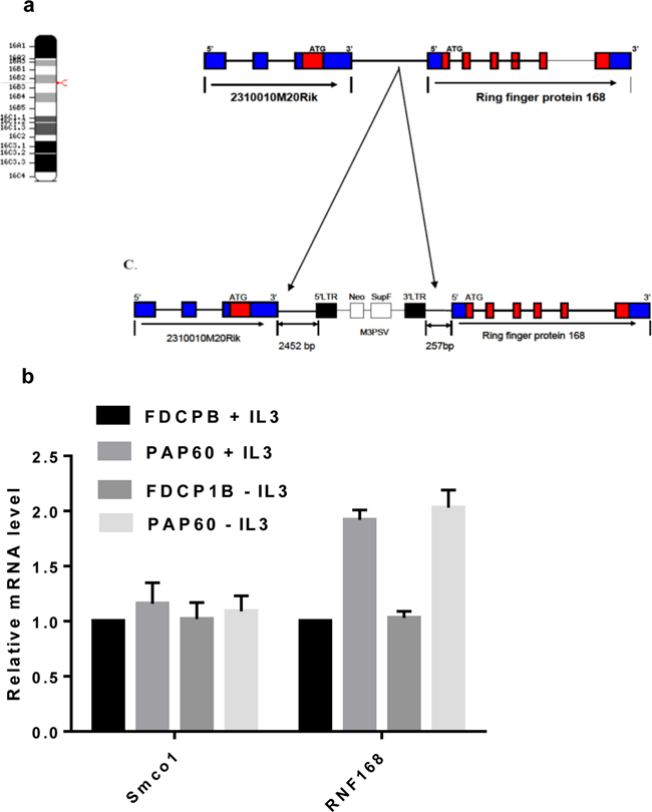
Identification of the provirus integration site in mouse chromosome 16 and expression of neighbouring genes (**a and c**) Schematic representation of mouse chromosome 16, Smco1 and RNF168. In the PAP60 cells, the provirus is integrated 229 bases upstream of the 5′ transcription start site of the RNF168 and 2452 bp downstream of the 3′ end of the *Smco1* gene. (**b**) Relative expression of Smco1 and RNF168 in FDCP-1B and PAP60 cells. PAP60 and FDCP1B cells were grown in the presence (1 ng/ml) of mIL-3 for 48 h. The cells were then washed and grown in the presence or absence of mIL-3 for 12 h. mRNA expression of Smco1 and RNF168 in parental FDCP-1B cells and PAP60 cells in the presence (1 ng/ml) or absence of mIL-3 were analysed by reverse transcription and real-time PCR with specific primers to Smco1, RNF168 and β-actin. The transcript levels are expressed relative to *β-actin* mRNA levels in the parental FDCP-1B cells. RT-qPCR measurements were repeated three times with similar results.

The key advantage of the RIM strategy is that it allows the site of insertion of the provirus to be determined by I-PCR [41,45]. This technique revealed that in the PAP60 cells, the provirus insertion site is in mouse chromosome 16, 229 bases upstream of the transcription start site of the gene encoding RNF168 (Figure 2a), and 2452 bases downstream of Smco1 (2310010M20Rik), a 200-amino acid single-pass membrane protein. The human homologue of Smco1 is a 214-amino acid protein known as C3orf43 [46,47]. Since the effects of proviral integration are not entirely predictable, the transcription levels of the two genes flanking the integration site were determined by RT-qPCR in PAP60 cells and the parental FDCP1B cells, both with and without IL-3 (Figure 2b). The level of expression of Smco1 was the same in PAP60 and parental FDCP1B cells. By contrast, the level of expression of RNF168 was doubled in PAP60 cells relative to parental FDCP1B cells. The expression of the two genes examined was unaffected by the presence of IL-3 in both PAP60 and parental FDCP1B cells (Figure 2b). Since the exact mechanism by which retroviral insertion increases transcription from the nearby *RNF168* gene is not clear, *RNF168* mRNA levels were manipulated in other ways (see below).

### Analysis of the role of RNF168 in mouse cells

**Figure 3:**
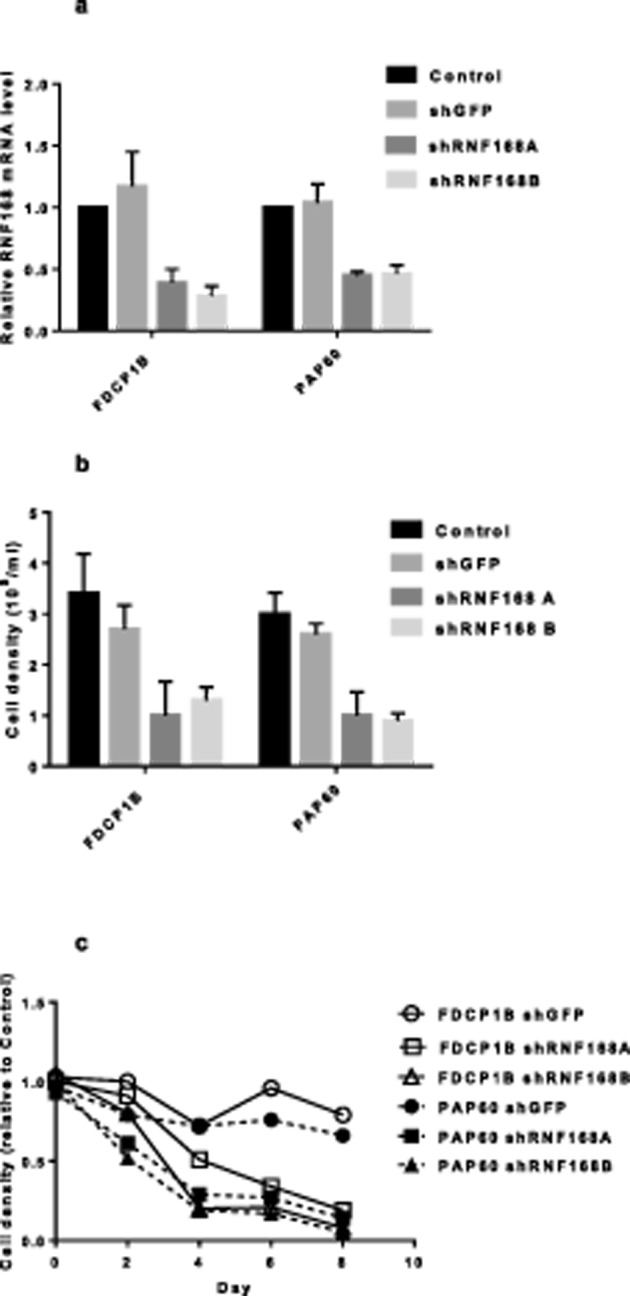
Down-regulation of RNF168 in FDCP-1B and PAP60 cells leads to reduced numbers of cells (**a**,**b**) FDCP-1B and PAP60 cells were transduced with LV-shRNF168A, LV-shRNF168B and LV-shGFP (MOI =3). (a) RNA levels and (b) cell density were determined on day 4 after transduction. Data are expressed as mean ± S.D., and statistical analyses of the data were performed using Student’s *t* test. Values of *P*<0.05 were considered to be statistically significant. The data are representative of three independent experiments for FDCP-1B and two independent experiments for PAP60 cells. (a) RNF168 RNA levels are expressed relative to the levels in the untreated corresponding cell line (PAP60 absolute level is two fold higher than FDCP1 absolute level Figure 2b). (**c**) Reduction in cell number by down-regulation of RNF168. FDCP-1B and PAP60 cells were transduced with LV-shRNF168A, LV-shRNF168B and LV-shGFP vectors, at an MOI of 3. Cell numbers were determined by MTT assay over an 8-day period. Data are expressed as a percentage MTT activity compared with non-infected cells.

Since the RT-qPCR analysis above suggested that RNF168 may be involved in the IL-3-independence observed in the PAP60 cells, the effect of down-regulation of endogenous RNF168 expression was examined. PAP60 and parental FDCP1B cells were transduced with shRNA vectors targeting RNF168. shRNAs targeting GFP were used as a control. RNF168-targeting shRNAs reduced *RNF168* mRNA levels by 60–70% in both PAP60 and FDCP1B cells (Figure 3a). This was accompanied by substantial reductions in culture cell density for both RNF168 shRNAs (Figure 3b), further implicating RNF168 in the control of proliferation in these murine growth factor dependent cells. This reduction in cell culture growth by both shRNF168 constructs was confirmed in a time-course experiment over 5 days (Figure 3c).

It is well established that RNF168 plays a key role in the repair of double-stand DNA breaks through the ubiquitination of nuclear proteins [23,28,33,48–50]. In order to examine the possibility that RNF168 might also be involved in modification of proteins in other cellular locations, Myc-tagged RNF168 was expressed in 293T cells and examined by fluorescence microscopy. RNF168 could only be detected within the nucleus, with a speckled appearance (Supplementary Figure S1). This nuclear distribution is consistent with the recently reported association of RNF168 with PML-NB [35].

### Analysis of the role of RNF168 in human haematopoietic cells

**Figure 4:**
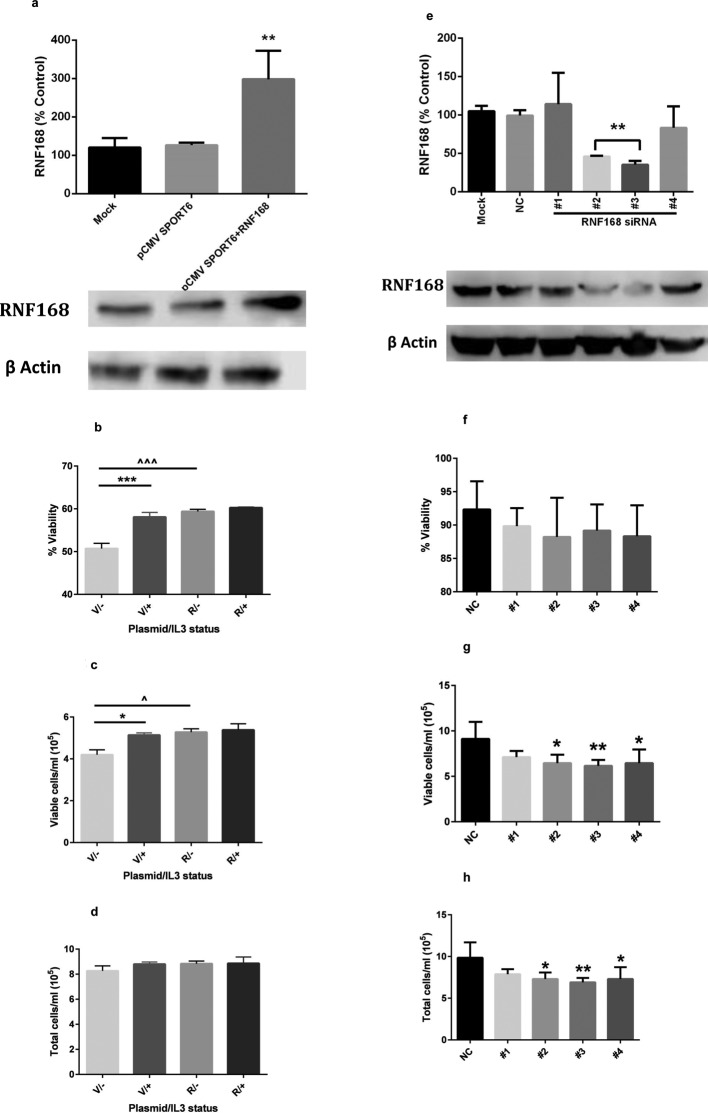
RNF168 regulates the growth and survival of the human growth factor dependent cell line, TF1 Cells were either transfected with a plasmid encoding human RNF168 or siRNA to human RNF168 and then cultured in the absence or presence of IL-3. (**a**–**d**) Cells were nucleofected with the plasmids pCMVSPORT6-RNF168 (R) or pCMVSPORT6 alone (V) and maintained in the presence of IL-3 for 20 h. (a) Western blot analysis demonstrating up-regulation of RNF168. Cells were then washed and replated at equal densities in medium containing (+) or without (–) IL-3 and cultured for a further 22 h before determining culture viability (b), viable cell number (c) and total cell number (d). Note that vector-transfected cells only demonstrate IL-3 dependence and that overexpression of RNF168 increases culture growth/viability in the absence of IL-3 only (*P <0.05, **P<0.01 and ***P<0.001 for intraplasmid comparisons; ^∧^*P*<0.05 and ^∧∧∧^*P*<0.001 for interplasmid comparisons; one-way ANOVA and Bonferroni’s MCT; *n*=5). (**e**–**h**) Cells were transfected with one of four different siRNAs to RNF168 (#1–#4) or with NC siRNA and maintained in the presence of IL-3 for 70 h. (e) Western blot analysis demonstrating down-regulation of RNF168. Cells were then washed and replated at equal densities in medium containing IL-3 and cultured for a further 70 h before determining culture viability (f), viable cell number (g) and total cell number (h). Note that silencing of RNF168 reduces culture growth (*P<0.05 and **P<0.01 compared with NC siRNA-transfected cells; one-way ANOVA and Dunnett’s MCT; *n* = 5).

In order to test the hypothesis that RNF168 might play a role in the regulation of growth factor dependence in human as well as mouse cells, we analysed the effect of overexpression of RNF168 in the human growth factor dependent cell line TF-1 [15]. TF-1 is a human haematopoietic cell line and TF-1 cells are dependent on IL-3 or GM-CSF for survival and proliferation [15]. TF-1 cells were transfected with a RNF168 expression construct, in order to overexpress RNF168, or with vector only (Figure 4a). Transfected cells were incubated in the presence or absence of IL-3 for 22 h; both culture viability and viable cell number in control vector transfected TF-1 cells were significantly reduced in the absence of IL-3. By contrast, culture viability and viable cell number were unaffected by IL-3 withdrawal in the RNF168 overexpressing TF-1 cells (Figure 4b–d), as seen for PAP60 cells (Figure 1), implicating RNF168 in the control of cell fate in human as well as mouse cells. Down-regulation of RNF168 in IL-3-supplemented TF-1 cells (Figure 4e) produced reductions in both the total cell density and the viable cell density of cultures (Figure 4f–h), further indicating a role for endogenous RNF168 in the proliferation of these human haematopoietic cells.

### Analysis of the role of RNF168 in human breast cancer cells

**Figure 5:**
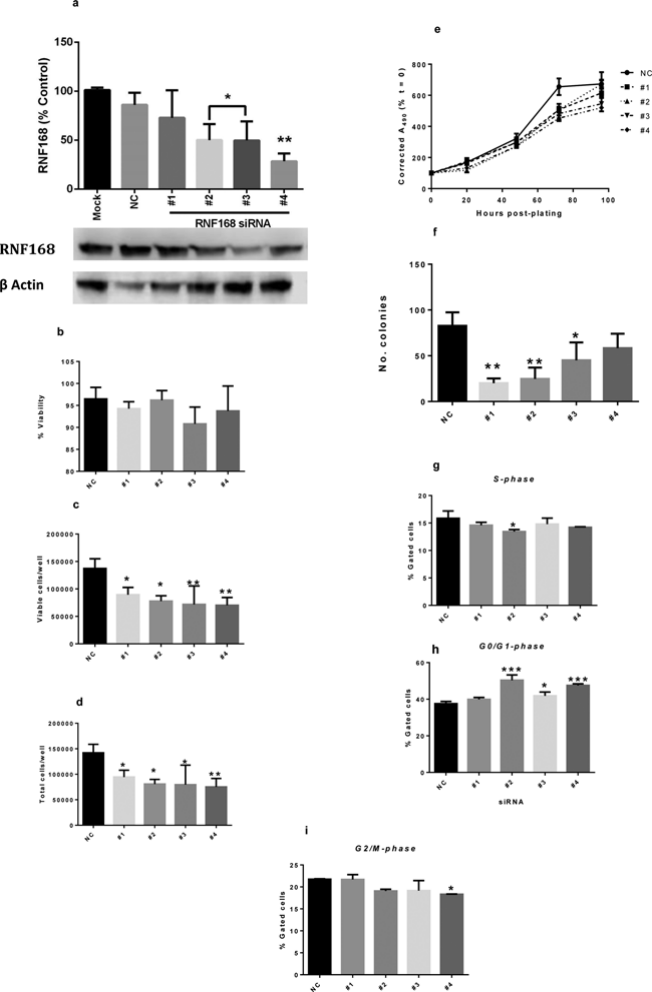
RNF168 also regulates the growth of human growth factor independent cells The breast cancer cell line, MCF7, was transfected with one of the four different siRNAs to RNF168 (#1–#4) or with NC siRNA and after 70 h, cells were replated at equal densities for analyses of culture growth and the cell cycle. (**a**) Western blot analysis demonstrating the effectiveness of the treatments. (**b**–**d**) Culture growth parameters determined by direct microscopic counting at 48 h *post*-replating. Note that silencing of RNF168 has negligible effect on culture viability (b) but significantly reduces viable (c) and total (d) cell numbers (*P<0.05 and **P<0.01 compared with NC siRNA-transfected cells; one-way ANOVA and Dunnett’s MCT; *n*≥3). (**e**) MTS assay of culture growth up to 96 h *post*-plating confirming reduced growth of cells transfected with siRNA to RNF168; statistical analysis (two-way ANOVA and Dunnett’s MCT; *n* ≥ 3) demonstrates different (*P*<0.05) values for all siRNAs at 72 h and for siRNAs #3 and #4 at 96 h. (**f**) Clonogenic assay data demonstrating that long-term growth of cells is compromised following silencing of RNF168 (*P<0.05 and **P<0.01 compared with NC siRNA-transfected cells; one-way ANOVA and Dunnett’s MCT; *n* ≥ 3). (**g**–**i**) Cell cycle analysis at 24 h *post*-replating, indicating reduced proliferation following silencing of RNF168 (*P<0.05, **P<0.01 and ***P<0.001 compared with NC siRNA-transfected cells; one-way ANOVA and Dunnett’s MCT; *n* = 3).

Since both the cell lines, mouse FDCP1B and human TF-1, are growth factor dependent haematopoietic cell lines, we extended the study to investigate the role of endogenous RNF168 in the proliferation and survival of human breast cell line MCF-7. MCF-7 cell cultures were treated with siRNAs specific for RNF168, and a non-targeting NC siRNA. Western blotting confirmed partial depletion of RNF168 protein (Figure 5a). RNF168 siRNAs significantly reduced MTS culture growth measurements and both viable cell density and total cell density (Figure 5b–e). In addition, RNF168 siRNAs substantially reduced the colony-forming ability of MCF-7 cells cloned for 72 h after treatment (Figure 5f). Cell cycle analysis indicated that depletion of endogenous RNF168 increased accumulation of cells in the G_0_/G_1_ phase, with corresponding decrease in cells in S-phase and G_2_/M (Figure 5g–i). These consistent observations indicate that the involvement of RNF168 in the control of human cell proliferation is not restricted to haematopoietic growth factor dependent cells.

## Discussion

A forward genetics strategy for the identification of functionally critical components of cell regulatory mechanisms has several important advantages: first, it is entirely independent of established knowledge; and second, it automatically focuses on elements that have controlling roles rather than on secondary phenomena [51–54]. RIM is an important application of the forward genetics strategy that has identified many unanticipated genes that encode key components of the mechanisms regulating cell fate. Many of these genes are implicated in oncogenesis and/or resistance to cancer therapies [18,19]. Our unbiased screen for genes implicated in growth factor independence in haematopoietic cells has identified an E3 ubiquitin ligase RNF168.

RNF168 has a well-established and important role in the modification of chromosomal proteins required for the repair of double-strand DNA breaks [23,28–32]. The observations presented above strongly suggest a broader role in the regulation of cell fate, i.e. in controlling cell survival and proliferation (Figures 1, 3–5). It is arguable that the amplification of *RNF168* in tumours is consistent with this wider role [32,34]. The observation that overexpression of RNF168 confers growth factor independence in both human and mouse haematopoietic cells (Figures 1, 3–5) suggests its involvement at key stages in pathways controlling cell fate. Moreover, our data suggest that this involvement of RNF168 in cell proliferation and survival is not limited to the cells of the haematopoietic lineage as reduction in the endogenous expression of RNF168 significantly affects the survival, proliferation and colony-forming ability of human breast epithelial cell line MCF-7.

Almost all previous reports of RNF168 activity have been concerned with the modification of nuclear proteins [31,55] and, consistent with this, our analysis of the subcellular localization of RNF168 (Supplementary Figure S1) indicates nuclear localization in patterns similar to those recently reported by Shire et al. [35]. In this context, it is important to appreciate that the post-translational modification of chromosomal proteins, including by ubiquitination [56,57], is involved in the regulation of many cellular processes, and this includes gene expression as well as DNA repair. For example, Bhatnagar et al. [27] reported that the ubiquitination of H2A by the E3 ubiquitin ligase TRIM37 resulted in the down-regulation of tumour suppressor genes in breast epithelial cells.

Several recent papers have shown that RNF168’s role extends beyond histone modification [35,58,59]. As well as PML [35], RNF168-mediated ubiquitination of FOXM1 (transcription factor forkhead box M1 [58] and TOP2α (topoisomerase IIα [59]) has also been demonstrated. The observation that RNF168 expression affects the accumulation of proteins in PML NBs may prove to be especially significant [35]. Down-regulation of RNF168 increased the level of PML NBs and overexpression of RNF168 produced a corresponding decrease in PML NBs [35]. PML is a well-established tumour suppressor [60–64] and a reduction or loss in expression of PML in other systems, e.g. in PML^−/−^ mice, results in increased proliferation and reduced apoptosis [60–63]. In particular, in TF1 cells, expression of the PML–RAR α fusion protein, which acts as dominant-negative PML [64], protects these cells from apoptosis induced by growth factor withdrawal, allowing growth factor independent proliferation. Negative regulation of PML by RNF168 [35] could therefore account for the inhibition of apoptosis and growth factor independent proliferation that we report here. The functional importance of RNF168 in regulating cell proliferation and apoptosis may have important implications for the cell’s response to DNA damage. Through connecting the DNA repair process to PML-NB activity, RNF168 may help to link the DDR to the subsequent fate of the cell.

In summary, an unbiased functional screen identified RNF168 in mouse haematopoietic cell lines that survived and proliferated in the absence of growth factor. Subsequent experiments suggest that RNF168 may play a key part of the mechanisms regulating cell survival and proliferation in both mouse and human cells, potentially through interaction with PML-NB.

## Supporting information

**Supplementary Figure 1.** Subcellular localization of RNF168. Myc-tagged wild-type (RNF168WT), RING domain mutated (RNF168H33A) and MIU domains mutated (RNF168A179G/A450G) RNF168 plasmids were transfected into 293T cells. RNF168 protein was visualized using anti-myc antibody; nuclei were counter stained with DAPI. RNF168WT (A) and RNF168H33A (B) proteins localized to nucleus with a dot-like, speckled pattern, while RNF168A179G/A450G (C) distributed evenly throughout the nucleus.

## Abbreviations

BRCA1: Breast Cancer 1
DDR: DNA damage response
hIL-3: human interleukin-3
IL-3: interleukin-3
I-PCR: inverse PCR
MCT: multiple comparison test
mIL-3: mouse interleukin-3
MOI: multiplicity of infection
NC: negative control
MoMuLV: Moloney Murine Leukemia Virus
PML: Promyelocytic Leukemia Protein
PML NB: PML nuclear body
RIM: retroviral insertional mutagenesis
RNF168: Ring Finger Protein 168
RT-qPCR: real-time quantitative RT-PCR
