# Supplementary material for: Retroviral insertional mutagenesis implicates E3 ubiquitin ligase RNF168 in the control of cell proliferation and survival
Source: Biosci Rep. 2017 Aug 14;37(4):BSR20170843. doi: 10.1042/BSR20170843 (PMC5634340; doi:10.1042/BSR20170843)

Supplemental Material For Publication

Supplementary Figure 1. Subcellular localization of RNF168. Myc-tagged wild-type (RNF168WT), RING domain mutated (RNF168H33A) and MIU domains mutated (RNF168A179G/A450G) RNF168 plasmids were transfected into 293T cells. RNF168 protein was visualized using anti-myc antibody; nuclei were counter stained with DAPI. RNF168WT (A) and RNF168H33A (B) proteins localized to nucleus with a dot-like, speckled pattern, while RNF168A179G/A450G (C) distributed evenly throughout the nucleus.

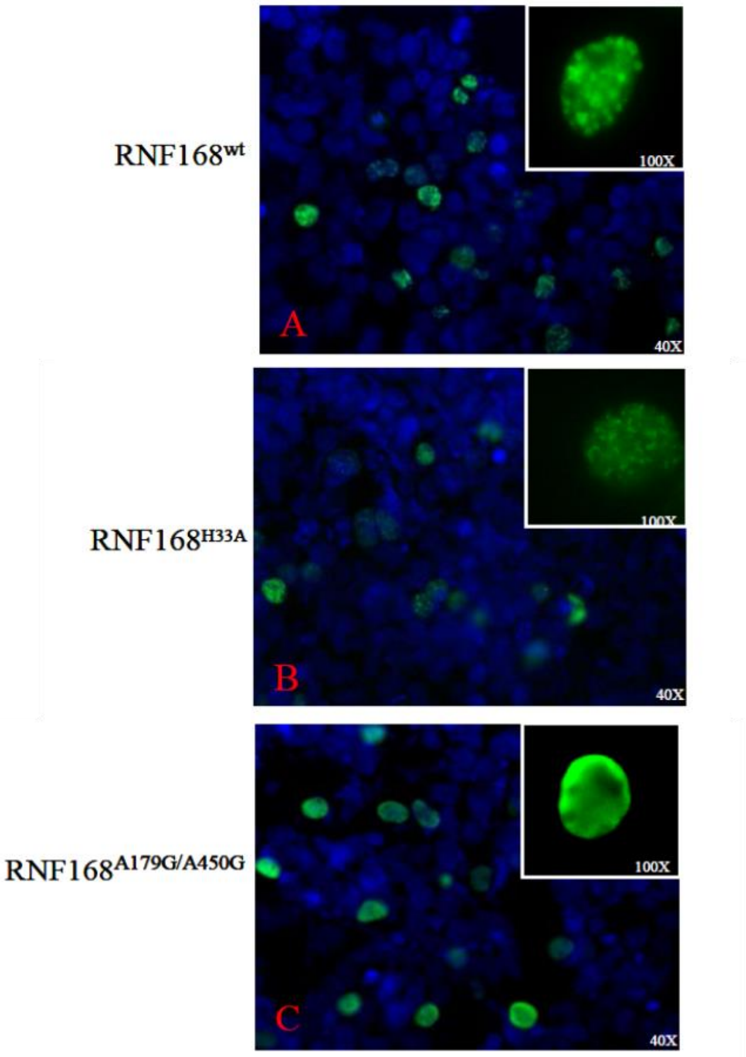

Supplement: Supplementary file 1 [file bsr-37-bsr20170843-s1.pdf]
